# Water Vapor Transfer and Near-Surface Salinity Contrasts in the North Atlantic Ocean

**DOI:** 10.1038/s41598-018-27052-6

**Published:** 2018-06-11

**Authors:** James Reagan, Dan Seidov, Tim Boyer

**Affiliations:** 1 Earth System Science Interdisciplinary Center, University of Maryland, College Park, MD USA; 2 National Centers for Environmental Information, NOAA, Silver Spring, MD USA

## Abstract

Maintaining North Atlantic (NA) intra-basin near-surface salinity (NSS) contrast between the high NSS (>37.0) in the subtropical NA (STNA) and low NSS (<35.0) in the subpolar NA (SPNA) has been shown to be important in sustaining the strength of the Atlantic Meridional Overturning Circulation. Evaporation (E) exceeding precipitation (P) in the STNA is primarily responsible for the high NSS there, whereas P dominating E in the SPNA contributes to its low NSS. With a basic understanding of NA intra-basin moisture transport, a correlation analysis was conducted between E-P/NSS over the NA subpolar gyre (SPG) and E-P across the rest of the NA over the 1985–2012 time period. Significant anti-correlations exist between E-P/NSS over the NA SPG and E-P over the central/northern STNA. This suggests that during times of high E over the central/northern STNA there is high (low) precipitation (NSS) over the SPG demonstrating a relationship likely exists between E over the STNA and NSS over the SPG. The maximum anti-correlated area is poleward of the maximum E-P location in the STNA, which is examined. These results provide a first step to ultimately utilizing NSS in the NA as a proxy for estimating changes in the hydrological cycle.

## Introduction

The North Atlantic (NA) is the saltiest of all ocean basins^1^ with strong and highly variable intra-basin sea surface salinity contrasts^2^, which are essential for the Atlantic Meridional Overturning Circulation (AMOC)^3,4^—the major element of the world ocean thermohaline circulation interconnecting all major ocean basins^5,6^. Very salty surface water in the subtropical North Atlantic (NA) is a prominent feature of near-surface salinity (NSS, here defined as the average salinity between 0 and 10 m depths) distribution, with salinity exceeding 37.0 (dimensionless on the PSS-78 Scale). These high NSS values in the NA subtropical gyre are due to evaporation (E) substantially exceeding precipitation (P)^7,8^.

Over 77% of global P and 85% of global E occurs over the ocean^7–9^. While most of P and E occurs over the ocean, their historical estimates are plagued by large uncertainties in satellite observations^10,11^, and assessing long-term change of E minus P (E-P) directly from measurements is therefore not feasible^12,13^. The satellite observation record (~1979-present) is also too short to assess long-term water cycle changes when compared to multi-decadal variability, and *in situ* data pre-1979 is full of large data gaps over the ocean^13^. Evaporation and precipitation, particularly the difference (E-P), has also been shown to be largely uncertain when assessing the global ocean water cycle using state of the art atmospheric reanalyses^14^. However, since global NSS changes are directly associated with E-P^15^, it is, in principle, possible to assess E-P indirectly through knowledge of the NSS variability^14,16–20^.

The AMOC functionality may be impacted by the long-term changes in NSS due to variability of the Earth’s water cycle which can modulate the high-latitude convective and mixing processes essential for the sinking branch of the AMOC^21^. It has been observed that salty regions of the ocean are becoming saltier and fresh regions fresher over the past ~40–60 years^22,23^ which has been directly related to an amplification of the global hydrological cycle^16,17,24^, albeit with varying rates^18^.

Through some estimates using multi-decadal climatologies of E and P^8^, the dominance of E over P yields a 1.16 Sverdrups (1 Sv = 10^6^m^3^sec^−1^) rate of freshwater loss in the Atlantic Ocean, whereas the dominance of P over E in the Pacific Ocean yields a rate of freshwater gain of 0.5 Sv across the sea surface. With E dominating the Atlantic and P dominating the Pacific, an amplification of this pattern would lead to further salinification of the Atlantic and freshening of the Pacific. This salinity inter-basin dipole has been amplifying since the 1950’s^17,22,23^ and is primarily sustained by ~0.5 Sv of water vapor over the subtropical Atlantic being transported across Central America and deposited as precipitation in the equatorial Pacific^25^. However, very little water vapor is transported from the Pacific and deposited as precipitation in the Atlantic due to the blocking of moisture transport by the Rocky Mountains^26^.

Models have shown that the inter-basin moisture transport—termed an “Atmospheric Bridge”^27^ —and resulting NSS contrasts between the Pacific and Atlantic is a key controlling mechanism of the global thermohaline circulation^27–29^. Additionally, this inter-basin salinity amplification is projected to continue^19^. Marsh *et al*.^27^ argued that the long-term fate of the AMOC is sensitive to two processes by which high Atlantic surface salinity is maintained, the Atmospheric Bridge and the Agulhas Leakage. In freshwater “hosing” model simulations^4,27,29,30^, high-latitudinal freshening and thus subtropical-subpolar NSS contrast in the NA have risen as an important if not key factor^3,4^ impacting the AMOC, while similar freshening in the Southern Ocean did not inflict noticeable AMOC change^4^.

The NSS intra-basin contrasts in the NA are especially important, as they are essential for the AMOC dynamics. Singh *et al*.^25^ showed that most of the P in the NA is sourced from the NA with very little moisture contribution from other ocean basins. To assess long-term ocean climate change, it is of paramount importance to understand how the salinity contrasts within the NA are being built and maintained. In our research we address one specific aspect of this broader task, namely—to uncover possible correlations between E-P and NSS in the subtropical and subpolar regions of the NA. The implications of detecting and quantifying such correlations may be far reaching—utilizing NA *in-situ* NSS observations as a proxy for estimating E-P.

Although the inter-basin water vapor exchange and inter-basin salinity contrasts are well documented, some ambiguity still exists regarding the connection between the intra-basin E and P distribution and salinity contrasts in all oceans. In more general terms, it is not yet well known how the spatial variability of the hydrological cycle correlates with the changes of the intra-basin NSS contrasts. This study aims to address this ambiguity.

## Discussion and Results

**Figure 1:**
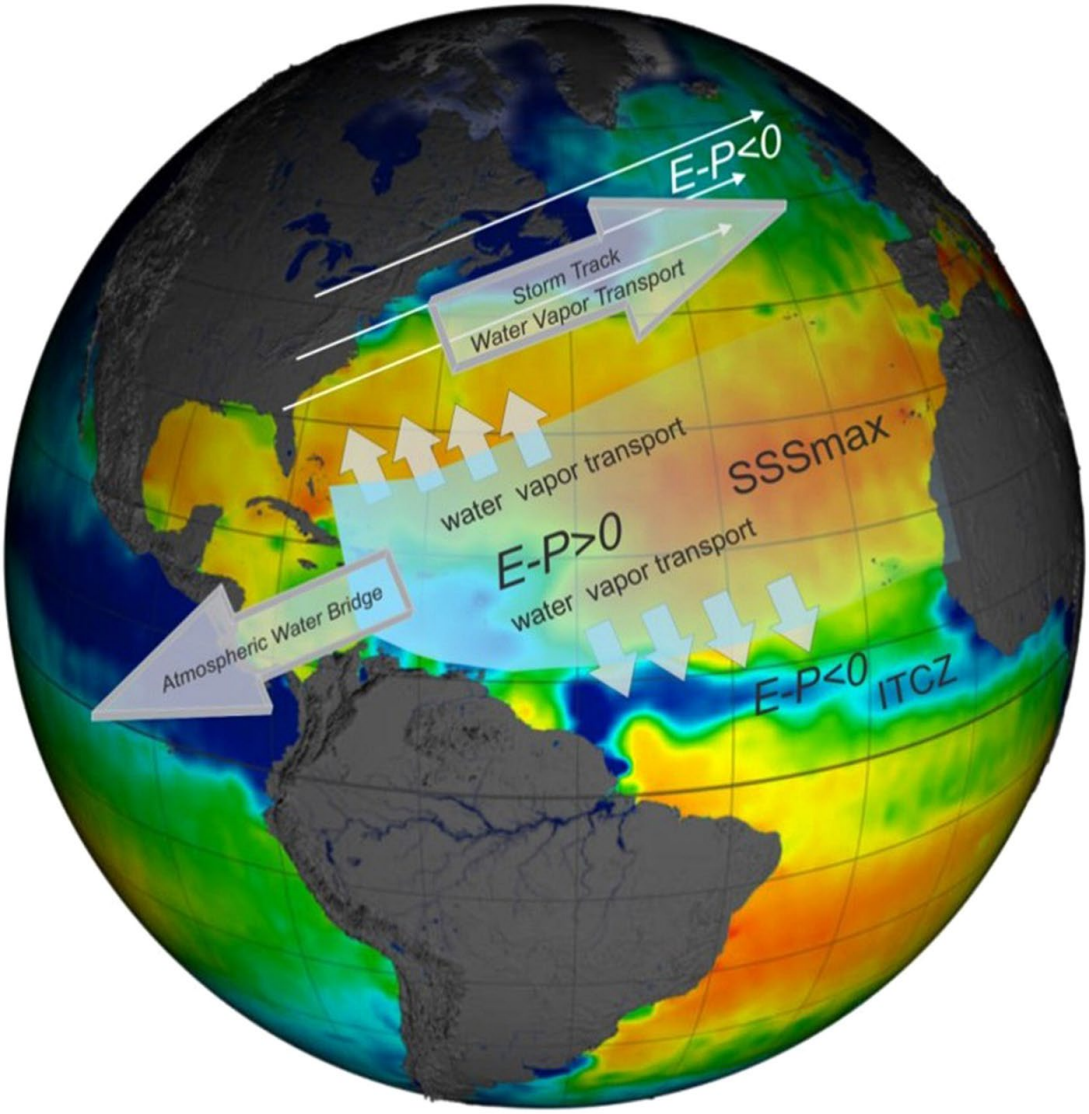
Schematic of the North Atlantic moisture transport. The background image with colored shadings of salinity is from the Aquarius satellite and courteous of NASA’s Goddard Space Flight Center Scientific Visualization Studio (https://svs.gsfc.nasa.gov/4046). Evaporation minus precipitation (E-P) is indicated by the white shadings, and atmospheric moisture transport is shown by the various arrows.

**Figure 2:**
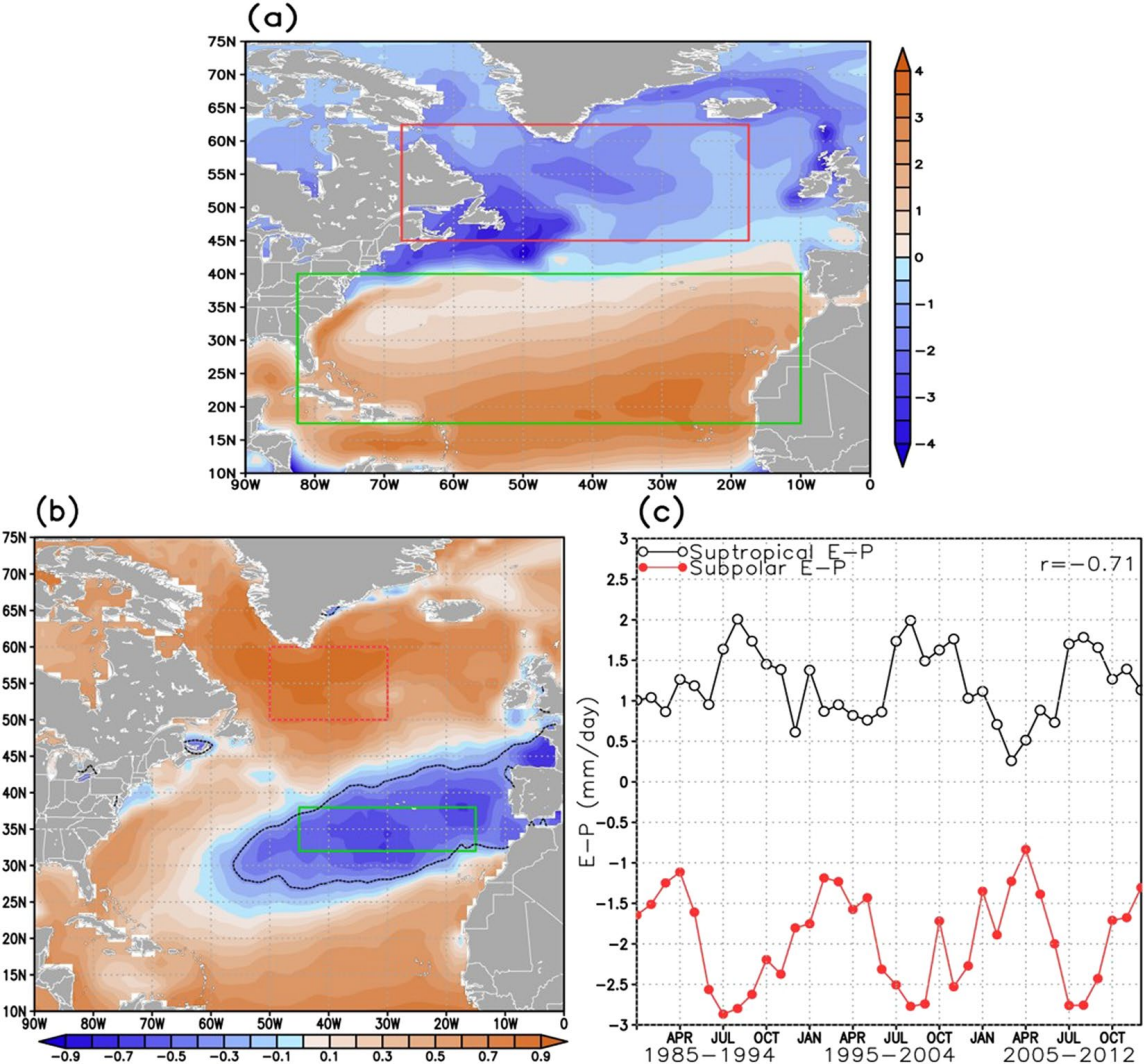
The 1985–2012 North Atlantic (**a**) Climatological E – P (mm*day^−1^), (**b**) correlation between the areal averaged subpolar gyre E-P (red-contoured rectangle in **b**) and the E-P over the rest of the North Atlantic Ocean, and **c**) time series of E-P over the subpolar NA (red box in **b**) and E-P over the subtropical NA (green box in **b**). Correlations and time series are based on the 1985–1994, 1995–2004, and 2005–2012 monthly climatological E and P fields (N = 36). The red box in (**a**) designates the subpolar North Atlantic (SPNA) region and the green box designates the subtropical North Atlantic (STNA). Black dotted line in (**b**) represents the region where correlation is lower than −0.330 (95% CI). This figure was created using the Grid Analysis and Display System (GrADS) software (available at: http://cola.gmu.edu/grads/).

The subtropical-subpolar NA atmospheric moisture transport is an effective and rapid (on the order of days to weeks) mechanism of freshwater transport between positive and negative E-P regions in the NA and is, along with advection of freshwater into the subpolar North Atlantic, one of the main candidates for maintaining the observed inter-gyre NSS contrasts. The mechanism of this moisture transport is schematically depicted in Fig. 1. Over the subtropical NA (STNA, approximate area shown in green box of Fig. 2a), where E > P, water vapor is produced and then diverges away from the NSS maximum salinity region. Although the strength and direction of the divergence of water vapor from the STNA varies with seasons^31,32^, in all four seasons it is captured and precipitated in four different regions: (i) along the Atlantic Intertropical Convergence Zone (ITCZ), (ii) in the eastern tropical Pacific ITCZ (via the aforementioned Atmospheric Bridge), (iii) off the East Coast of North America, and (iv) in the subpolar North Atlantic (SPNA, approximate area shown in red box of Fig. 2a). Since the ITCZ dynamics and potential impacts on tropical NSS is outside our focus on the extratropical NSS interconnections, only the SPNA and STNA NSS contrasts and their relations to the hydrological cycle are the subject of this study.

In the atmosphere, water vapor transported to the East Coast of North America is typically captured within eastward/northeastward moving mid-latitude cyclones that generally originate due to baroclinic instability caused by sharp temperature gradients off the East Coast of North America. These storms are stronger and more frequent in winter due to increased baroclinic instability. They generally follow a similar northeast trajectory (i.e., storm tracks) directing them and their associated precipitation to the subpolar and northeast Atlantic regions^33–36^. Excess E over P along the Gulf Stream path and in the STNA also provides moisture for NA atmospheric rivers^37^ throughout the year^38^. Alternatively, a north/northwestward moisture transport from the E > P STNA region (see the schematics in Fig. 1) can cause convergence in the SPNA leading to precipitation, particularly during certain seasons.

There are two major pathways for water vapor produced in the STNA to be transported and deposited (as precipitation) in the SPNA: the indirect pathway (i.e., water vapor is captured in a mid-latitude cyclone and transported along the NA East Coast storm track) and the direct pathway (i.e., north/northwestward moisture divergence from STNA with convergence over SPNA), as the scheme in Fig. 1 suggests. For the indirect pathway, the latency between water vapor production in the STNA and deposition in the SPNA is at most a week or two, with the direct pathway requiring just several days.

The rate of freshening in the subpolar gyre (SPG) depends on the amount of water vapor transported northward from the subtropical gyre (STG) that precipitates over the SPG leading to P dominating E. For our analysis, we computed the monthly climatological distribution of evaporation minus precipitation (E-P) averaged over the time period between 1985 and 2012 (Fig. 2a) using the Objectively Analyzed air-sea Fluxes^39^ (OAFlux) monthly evaporation fields and the Global Precipitation Climatology Project version 2.2^40^ (GPCP) monthly precipitation fields (full description and reasoning of data selection, data processing, and calculations can be found in Methods). The maximum of E-P (>4.0 mm/day) occurs in the southeast NA, between 15°N and 40°N centered around 30°W and 20°N. There is also a secondary maximum along the US east coast following the Gulf Stream path. E-P slowly decreases to the north, with P beginning to exceed E at about 40°N and with the maximum negative E-P (<−4.0 mm/day) occurring southeast of Newfoundland. On average, the SPNA has E-P rates of roughly −2.0 mm/day.

To assess the STNA-SPNA moisture connection, a geographic E-P correlation analysis was performed using the OAFlux evaporation and GPCP precipitation fields. Figure 2b represents the correlations between the area-averaged E-P in the SPG (subset region of SPNA identified as the red rectangle in Fig. 2b) and E-P throughout the rest of the NA over the three decadal time period from 1985–2012 (1985–1994, 1995–2004, and 2005–2012). High positive correlations (>0.5) exist in not only the SPG (where the area-averaged E-P was calculated), but also along the Gulf Stream path. High anti-correlations (<−0.5) extend from the central STNA to the northeast STNA, along the region of decreasing E-P (as shown in Fig. 2a). This is consistent with other reanalyses and hybrid E-P products (see Supplementary Fig. S1). Hence, when E-P increases (i.e., increased water vapor production) in the central and northeastern regions of the STNA, E-P decreases (i.e., increased precipitation) in the SPG. This can be clearly seen in Fig. 2c showing the area-averaged 36-month time series of E-P for the subpolar (red box in Fig. 2b) and subtropical (green box in Fig. 2b) regions. The full time series has a −0.71 correlation (p < 0.0001), with late boreal summer/early fall being most noticeably anti-correlated. Other reanalyses and hybrid E-P products show similarly strong anti-correlated time series of intra-basin E-P (see Supplementary Fig. S2). During this time, the northern subtropics experience maximum E-P (i.e., maximum evaporation), while the SPG has minimum E-P (i.e., maximum precipitation). Additionally, the positive correlation between E-P over the Gulf Stream path and the SPG E-P (Fig. 2b) indicates that they share a similar seasonal cycle of precipitation, which is likely due to their strong connection through the storm tracks as shown in Fig. 1. Essentially, Fig. 2 implies that the water vapor produced over the central and northeastern STNA is transported both directly and indirectly to the SPG where it is deposited as precipitation.

The E-P relationship between the STNA and SPNA is rather straightforward; E-P is primarily based on the convergence/divergence of vertically integrated moisture fluxes^41^. NSS, on the other hand, is affected by many other factors besides E-P^42^, but understanding the SPNA NSS relationship with the STNA E-P is essential to eventually estimating E-P from NSS (or vice-versa) in the North Atlantic. This first step towards a better understanding of intra-basin relations between E-P and NSS can be viewed as a starting point for future work on utilizing subpolar NSS measurements as a proxy for estimating NA E-P.

**Figure 3:**
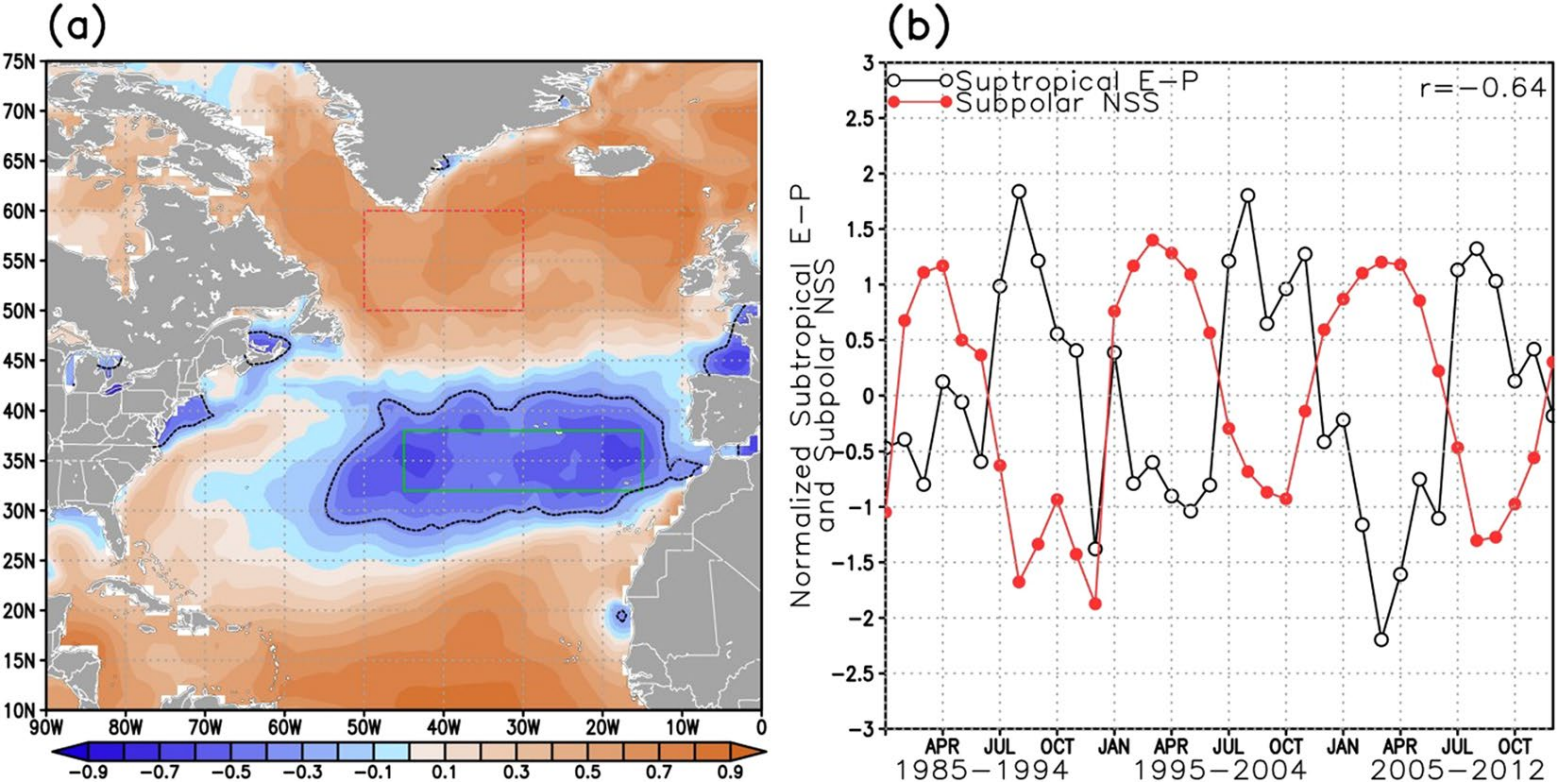
Similar to Fig. 2, but (**a**) represents correlation between area-average subpolar NSS (red box) and NA E-P and (**b**) is the normalized time series of NSS from the red box in (**a**) and E-P from the green box in (**a**). Black dotted line in (**a**) represents the region where correlation is lower than −0.330 (95% CI). This figure was created using the GrADS software.

The correlation between the SPG NSS (SPG-NSS) and NA E-P (Fig. 3a) shows a pattern similar to the SPG E-P and NA E-P correlation (Fig. 2b). All NSS calculations are based on the World Ocean Atlas 2013^1^ (WOA13) fields. The positive correlations of E-P (~0.4–0.6) in the SPG with the area averaged SPG NSS (red box in Fig. 3a) illustrates that local E-P plays a role in the near-surface salinity budget (NSSB) in the subpolar area, but E-P may not dominate all other terms in the NSSB (i.e., advection, meltwater, entrainment, etc.)^42^. This is especially so during certain seasons (e.g., during spring when sea ice melts and thus augments E-P in freshening the sea surface). There is a large E-P area that extends from the northcentral STNA to the northeastern STNA where the SPG NSS and E-P are significantly (95%, r < −0.330) anti-correlated (Fig. 3a). The pattern in this region is very similar to the area with significant anti-correlation in Fig. 2b. Thus, when there is high E-P in the northcentral to northeastern STNA region, there is low NSS in the SPG.

A time series in both areas may help better illustrate these inter-relations. Figure 3b shows the same area-averaged regions of Fig. 2b (green box = northern subtropics, red box = SPG), but plots SPG NSS rather than E-P. The SPG NSS and the STNA E-P 36-month time series were then normalized to allow plotting on the same figure (Fig. 3b). During late summer/early fall when E-P is at maximum in the northern subtropics, NSS is at its lowest in the SPG. The correlation is −0.64 (p < 0.0001) between the area-averaged NSS of the SPG and E-P over the northern subtropics. This means that the additional water vapor produced in the northcentral/northeastern STNA region during high E-P periods is likely transported, via both direct and indirect pathways, to the SPG where it falls as precipitation lowering NSS. The NSS and E-P do not anti-correlate quite as strongly as seen in Fig. 2b because NSS in the SPG is shaped by more than just local E-P as discussed previously^42^. The region of highest anti-correlations between SPG NSS and NA E-P in Fig. 3a and the corresponding area-averaged time series in Fig. 3b are consistent with other reanalyses and hybrid E-P products (see Supplementary Figs S3 and S4), with some reanalyses extending the area of significant anti-correlations across the entire North Atlantic (Supplementary Fig. S3).

**Figure 4:**
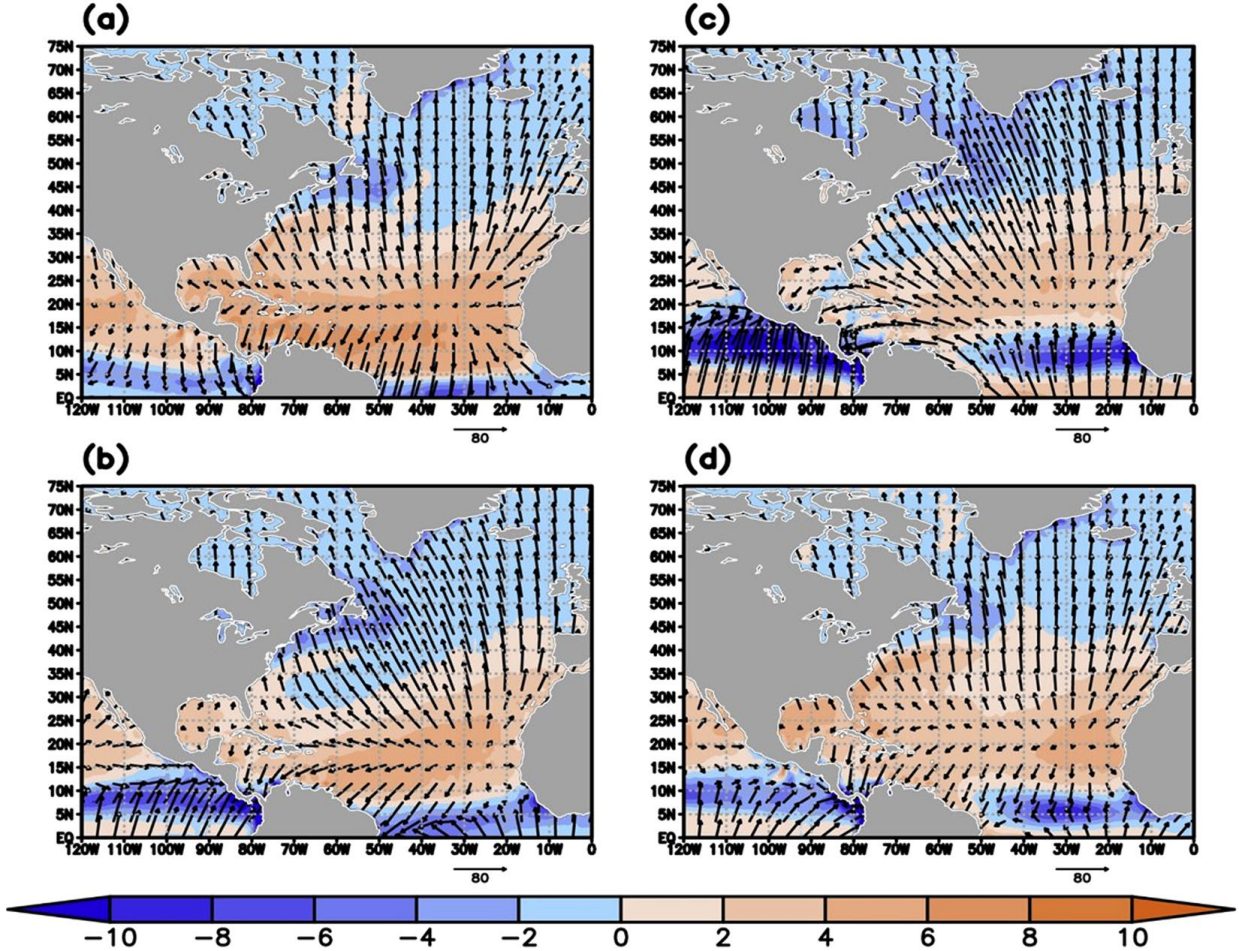
The 1985–2012 seasonal average of the vertically integrated moisture flux divergence (VIMFD, mm*day^−1^, shaded) and the divergent component of the moisture fluxes (DCMF, kg*m^−1^*sec^−1^, vectors) for (**a**) winter (JFM), (**b**) spring (AMJ), (**c**) summer (JAS), and (**d**) fall (OND). Orange shades represent moisture divergence and blue shades represent moisture convergence. This figure was created using the GrADS software.

The highest anti-correlations in Fig. 3a are to the north (latitude range of ~30°–40°N) of the E-P maximum in the STNA (see Fig. 2a). While this may be counter-intuitive, the 1985–2012 seasonal averages of the divergent components of the vertically integrated moisture flux (DCMF) vectors over the NA (Fig. 4a–d, black vectors) from the Modern-Era Retrospective analysis for Research and Operations – Version 2 (MERRA-2)^43^ imply that the anti-correlation in Figs 2b and 3a are located in regions where the divergent component of the moisture flux is directed poleward away from the region of maximum E-P. Additionally, the vertically integrated moisture flux divergence (VIMFD) is slightly positive (Fig. 4a–d, orange shades) but becomes negative (Fig. 4a–d, blue shades) at the northern extent of this STNA region characterized by significant anti-correlations. The divergent components of the moisture fluxes converge (Fig. 4a–d, blue shades) in the SPNA with additional convergence in spring and summer along the storm track area in the STNA. This is generally consistent with other reanalyses (see Supplementary Figs S5 and S6). In a statistically steady state of the atmosphere, and more general 2-D formulation, E-P is balanced by the divergence of the horizontal moisture flux, *E*−*P*=**∇**·**F**, where **F** is the vector of horizontal moisture flux integrated over the depth of the atmosphere^41^, thus the convergence (divergence) regions in Fig. 4a–d indicate where P (E) exceeds E (P). It should be noted that there exists high anti-correlations between E-P along the near-coast of North America and SPG NSS (Fig. 3a). They are likely due to unrelated anti-correlated seasonal cycles as there is little to no moisture production (Fig. 4a–d, orange shades) in this region that can be transported to the SPG, and thus no easily seen physical mechanism linking the two. Additionally, the DCMF (Fig. 4a–d, vectors) in this region is mainly north-northwestward and not directed towards the central SPG.

**Figure 5:**
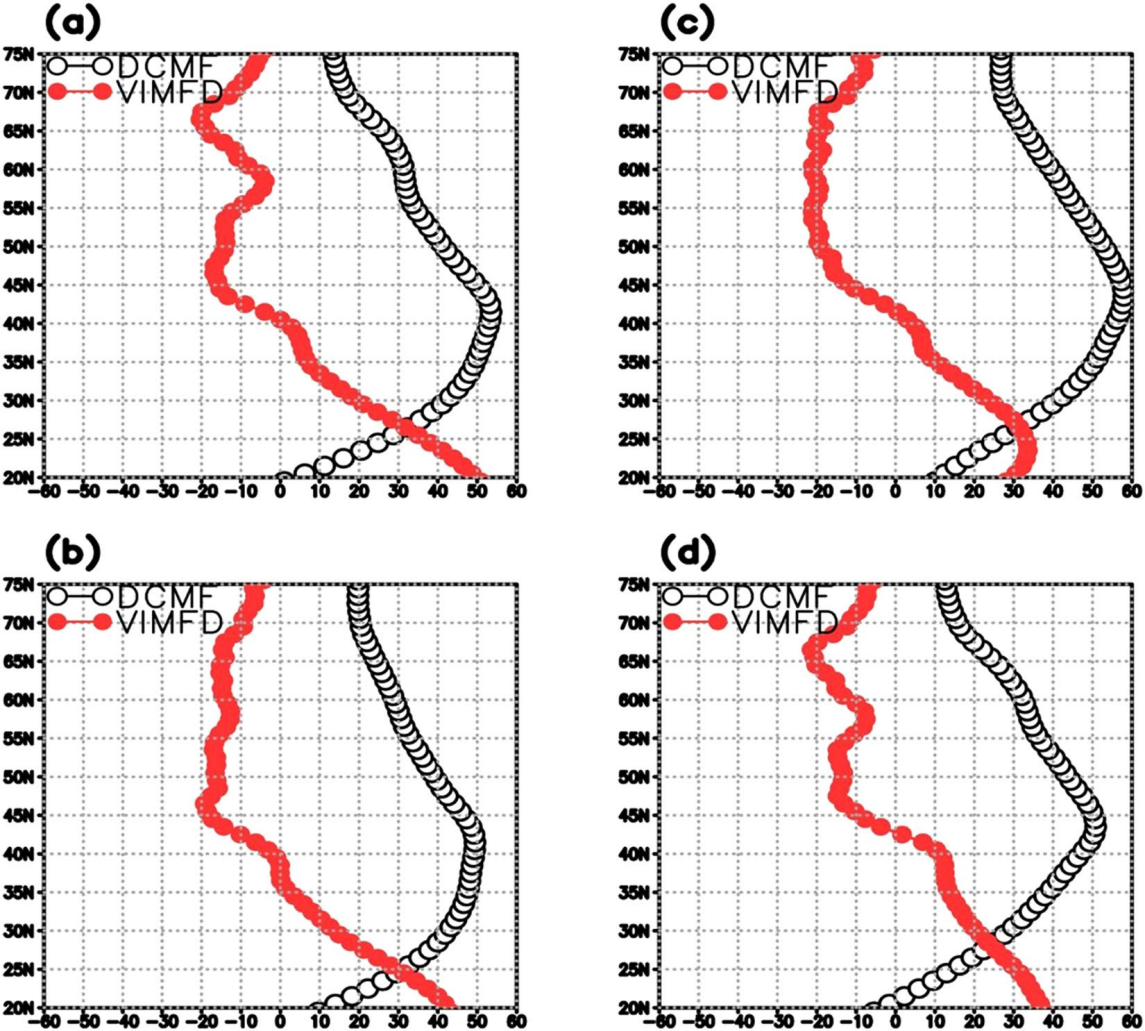
The 1985–2012 zonal average of the meridional component of the DCMF (kg*m^−1^*sec^−1^, black line) and of the VIMFD (10*mm*day^−1^, red line) for (**a**) winter (JFM), (**b**) spring (AMJ), (**c**) summer (JAS), and (**d**) fall (OND). The zonal average was taken over the 60°W–20°W area (see Fig. 4a–d). This figure was created using the GrADS software.

Examination of the meridional component of the divergent moisture fluxes (see Fig. 4a–d, vectors) demonstrate that, after taking the zonal average across 60°W–20°W over the 20°N–75°N domain, the poleward divergent moisture fluxes are strongest between 35° and 45°N for nearly all seasons (Fig. 5a–d, black line). Importantly, these poleward divergent fluxes are strongest (~60 kg*m^−1^*sec^−1^) in summer (Fig. 5c, black line), coinciding with the highest E-P in the northern subtropics (Figs 2c and 3b). Additionally, the zonal average of the vertically integrated moisture flux divergence (Fig. 5a–d, red line) shows that the strongest convergence (~−2 mm*day^−1^) in the subpolar regions (50°–60°N) also occurs in late summer (Fig. 5c, red line). These findings are consistent with another state-of-the-art reanalysis and differ somewhat from an older generation reanalysis (see Supplementary Figs S7 and S8). However, the moisture fluxes from all products agree on the significant impact that the meridional component of divergent moisture fluxes can have on the redistribution of moisture in the North Atlantic and the role it can play in sustaining the NSS inter-gyre contrast.

Bringing all analyses together, it can be concluded that during late summer when E-P is at its maximum in the northern subtropics (Fig. 2c- black line) there are near-maximum poleward divergent moisture fluxes (Fig. 5c, black line) occurring in the vicinity at the same time. The poleward transport of this excess moisture yields ample amounts of available precipitable water, and with maximum convergence also occurring in summer (Fig. 5c, red line) over the SPG we see this moisture fall as precipitation in the SPG leading to lower NSS there (Fig. 3b, red line). This seasonal cycle explains why such strong anti-correlations exist between E-P over the subtropics and E-P/NSS over the SPG.

## Conclusions

The NA intra-basin NSS contrast and intra-basin moisture transport are connected and may play an important role in maintaining the AMOC functionality. Although this focal point in ocean climate dynamics has been intensely investigated (see recent review^21^), the connection between NA intra-basin water vapor production, transfer, and deposition and the inter-gyre NSS contrast remained largely underexplored. The provided correlation analysis in this study between NSS and E-P over the NA aims at improving the understanding of the role the hydrological cycle plays in maintaining the NA inter-gyre NSS structure. The major conclusions are the following:

1. Subtropical E-P is significantly (95%, r < −0.330) anti-correlated with subpolar E-P in the NA and is consistent with multiple hybrid and reanalysis E-P products. The strong anti-correlations occur mainly between 30° to 40°N in the subtropics, which is also near the area where the poleward divergent component of the moisture fluxes are strongest. It implies that a significant amount of water vapor produced in the northcentral and northeastern STNA is transported poleward and deposited as precipitation in the SPG inferring substantial extra-tropical intra-basin redistribution of freshwater.
2. The same processes cause significant anti-correlations (95%, r < −0.330) between the subtropical E-P and subpolar NSS in the NA implying the seasonal cycle of E-P (particularly summer) over the northern subtropical NA and subpolar NSS are connected through intra-basin moisture transports.
3. In late summer, the E-P is at its maximum over the northern subtropical NA, and so is the strength of the poleward-directed moisture fluxes. This allows ample moisture to be pumped poleward where it converges over the subpolar region (also reaching maximum in summer) and precipitates. Subsequently, the precipitation freshens the near-surface leading to decreased NSS.

In summary, the intra-basin moisture transport from the STNA is immensely important for maintaining intra-basin NSS contrasts. Future work in utilizing observed subpolar NSS as a proxy for estimating historical and current changes in the hydrological cycle is needed as there is a clear and coherent relationship between those two. Additionally, if the estimates of E over P substantially improve, it may permit the usage of E-P estimates over the subtropical NA to forecast changes in subpolar NSS, which can carry many different and far fetching implications.

## Methods

This study operates with four different variables of the ocean-atmosphere system in the North Atlantic Ocean: near-surface salinity, evaporation, precipitation, and moisture fluxes. For each of these variables, monthly decadal climatologies were calculated for the 1985–1994, 1995–2004, and 2005–2012 decades. For moisture fluxes, in addition to the three aforementioned monthly climatologies, seasonal climatologies were also calculated.

All gridded data used in this study and in the Supplementary Information were interpolated to a 1° × 1° spatial grid. For original data that was of a finer resolution than 1° × 1°, the data were interpolated using box-averaging. For original data that was of a coarser resolution than 1° × 1°, the data were interpolated using bilinear interpolation. Salinity data is a dimensionless quantity reported on the PSS-78 Scale. All evaporation, precipitation, and vertically-integrated moisture divergence data were converted to units of mm*day^−1^. Moisture fluxes were, if necessary, converted to kg*m^−1^*sec^−1^. For all fields, with the exception of salinity which is already available as monthly decadal climatologies, the decadal monthly climatologies were calculated from the monthly fields by averaging all months within each decade of interest. For example, the January 1985–1994 evaporation decadal climatology was compiled by averaging the monthly evaporation from January 1985, January 1986, and so on through January 1994. Thus, all decadal climatologies computed and used in this study were presented on the same temporal and spatial resolutions. Below is a brief description of all datasets used and where they can be acquired.

### Salinity

The monthly decadal climatological salinity data are from the World Ocean Atlas 2013 Version 2 (WOA13)^1^ (available at: https://www.nodc.noaa.gov/OC5/woa13/). The WOA13 salinity fields were calculated using *in situ* profile data from the World Ocean Database^44^. The WOA13 provides gridded climatological salinity fields for the 1955–1964, 1965–1974, 1975–1984, 1985–1994, 1995–2004, and 2005–2012 decades as well as an average of those six decades. For this study, only the monthly fields for the last three decades (i.e., 1985–1994, 1995–2004, and 2005–2012) were used. Additionally, near-surface salinity was defined to be the average of the first three levels (i.e., 0, 5, and 10 m) of the WOA13 salinity fields. The 0–10 m average salinity was used rather than just the 0 m salinity for two reasons: (1) mixed layer depth in the subpolar region is generally greater than 10 m throughout the year^45^ yielding well-mixed salinity in the 0–10 m depth range and (2) to ensure instruments whose shallowest measurements are made at depths greater than 5 m (i.e., many Argo floats shallowest measurements are made between 5–10 m) are included in our analysis.

### Evaporation

The evaporation data is from the Woods Hole Oceanographic Institute (WHOI) Objectively Analyzed air-sea Fluxes (OAFlux) Project^39^ (available at: http://oaflux.whoi.edu/index.html). The original evaporation data are monthly from January 1958 through September 2015 in units of cm*yr^−1^.

For the Supplementary Information, additional evaporation products were used and underwent similar transformations to establish the decadal climatologies. These products include the following reanalyses: the European Centre for Medium-Range Weather Forecasts (ECMWF) Reanalysis-Interim^46^ (ERA-I), the Modern-Era Retrospective analysis for Research and Applications – Version 2^43^ (MERRA-2), and the NCEP/NCAR Reanalysis 1 Project^47^ (NCEP). Additionally, we also utilized the evaporation data from the Coordinated Ocean-ice Reference Experiments version 2^48^ (COREv2).

### Precipitation

The precipitation data is from the Global Precipitation Climatology Project (GPCP)^40^ (available at: https://www.esrl.noaa.gov/psd/data/gridded/data.gpcp.html). For this study, version 2.2 of the data was used. The original precipitation data were monthly 2.5° × 2.5° fields from January 1979 through September 2015 in units of mm*day^−1^.

For the Supplementary Information, additional precipitation products were used and underwent similar transformations to establish the decadal climatologies. These products include the following reanalyses: ERA-I, MERRA-2, and NCEP. Additionally, we also utilized the COREv2 precipitation data. The COREv2 precipitation data blends multiple precipitation products, some of which merge *in situ* and satellite observations. Additionally, the evaporation data in COREv2 is, at least partially, derived from variables provided by reanalysis output^48^. Therefore, because the COREv2 E-P dataset is a mixture of *in situ* observations, satellite observations, and reanalysis data, we consider it a hybrid E-P product in this study.

### Evaporation minus Precipitation (E-P)

We combine the OAFlux evaporation and GPCP precipitation fields to assemble E-P. These selected products have been successfully utilized in many recent studies relating ocean salinity and the water cycle^14,17,18,20,42,49^. Additionally, the OAFlux evaporation and GPCP precipitation were also found to be in good agreement with *in situ* measurements and, when combined with river runoff data, do a good job at closing off the oceanic freshwater budget^11^.

### Moisture Fluxes

The zonal and meridional moisture flux data is from the Modern-Era Retrospective analysis for Research and Operations – Version 2^43^ (MERRA-2) reanalysis. The data is available at: https://gmao.gsfc.nasa.gov/reanalysis/MERRA-2/data_access/. The original MERRA-2 moisture fluxes were monthly 0.5° × 0.625° in units of kg*m^−1^*sec^−1^. One of the primary focuses of MERRA’s (MERRA-2’s predecessor) development was an improved representation of the global water cycle, and that focus continued with MERRA-2’s development^43,50^, which justified our selection of this product for moisture flux related analysis.

For the Supplementary Information, additional moisture fluxes were used and underwent similar transformations to establish the decadal climatologies. These products include the following reanalyses: ERA-I and NCEP.

The ERA-I and NCEP evaporation minus precipitation and moisture flux data were made available through the National Center for Atmospheric Research (NCAR) Climate and Global Dynamics (CGD) Climate Analysis Section’s Data Catalog (available at: http://www.cgd.ucar.edu/cas/catalog/index.html). The COREv2 evaporation and precipitation data were made available through the Research Data Archive at the National Center for Atmospheric Research (available at: https://rda.ucar.edu/datasets/ds260.2/).

For NSS, E, and P analyses, we only used the three decadal climatologies spanning from 1985 through 2012. While there is NSS, E, and P data predating this time period, the launch of microwave imaging satellites in 1987 allowed for improved estimates of certain water flux parameters (e.g., wind speed), thus a discontinuity in the E and P data arises around 1987^11^. Our earliest decade begins in 1985 which is pre-1987; however, we see little difference in the E and P fields whether we use 1988–1994 or the 1985–1994 period, likely due to the large amount of averaging that the data undergoes to form decadal climatologies. Some atmospheric reanalyses do contain E, P, and moisture flux fields before the satellite era (~1979), but the lack of *in situ* data before 1979, particularly over the oceans, causes a severe lack of data being assimilated into the reanalysis models. Supplementary Fig. S9 illustrates the area-averaged monthly decadal averages of E-P over the 1957–2002 period from the ERA-40 reanalysis^51^ in the subpolar and subtropical NA. There is a clear degradation of the seasonal cycle of E-P during the 1957–1964 and 1965–1974 periods (as compared to the latter 3 decades) over the subpolar NA, likely due to the misrepresentation of precipitation in the subpolar NA. Thus, we confine this study to the 1985–2012 period. The ERA-40 data was acquired from the Asia-Pacific Data-Research Center (http://apdrc.soest.hawaii.edu/data/data.php).

The correlation calculations performed in this study and additional results provided in the Supplementary Information were done through calculating the area-average E-P and NSS in the subpolar gyre (we defined the subpolar gyre as the area encompassing 50°–30°W and 50°–60°N), and correlating the 36 month time series (3 decadal monthly climatologies) to each grid point’s E-P time series in the NA. Additionally, we computed the area-average E-P over the northern subtropics (defined as the area encompassed by 45°–15°W and 32°–38°N) to be used in the time series plots and was also used to compute the time series correlations with area-average SPG NSS and E-P. For all correlation calculations, the data were de-trended using simple linear regression. The sample size (i.e., time period) for each correlation calculation is 36 (N = 12 months * 3 decades) with 34 degrees of freedom (N-2).

## Electronic supplementary material

Supplementary Information

## References

[1] Zweng, M. *et al*. World Ocean Atlas 2013, Volume 2: Salinity. NOAA Atlas NESDIS 74 (2013).

[2] Reverdin G, Kestenare E, Frankignoul C, Delcroix T (2007). Surface salinity in the Atlantic Ocean (30°S–50°N). Progress in Oceanography.

[3] Seidov, D. & Haupt, B. J. Freshwater teleconnections and ocean thermohaline circulation. *Geophysical Research Letters* **30**, 10.1029/2002GL016564 (2003).

[4] Stouffer RJ, Seidov D, Haupt BJ (2007). Climate Response to External Sources of Freshwater: North Atlantic versus the Southern Ocean. Journal of Climate.

[5] Broecker WS (1991). The Great Ocean Conveyor. Oceanography.

[6] Gordon AL (1986). Interocean exchange of thermocline water. Journal of Geophysical Research: Oceans.

[7] Schmitt RW (1995). The ocean component of the global water cycle. Reviews of Geophysics.

[8] Schmitt, R. W. Salinity and the Global Water Cycle. *Oceanography* **21**, 10.5670/oceanog.2008.63 (2008).

[9] Durack, P. J. Ocean Salinity and the Global Water Cycle. *Oceanography* **28** (2015).

[10] Lagerloef, G., Schmitt, R., Schanze, J. & Kao, H.-Y. The Ocean and the Global Water Cycle. *Oceanography* **23**, 10.5670/oceanog.2010.07 (2010).

[11] Schanze JJ, Schmitt RW, Yu LL (2010). The global oceanic freshwater cycle: A state-of-the-art quantification. Journal of Marine Research.

[12] Robertson FR (2014). Consistency of Estimated Global Water Cycle Variations over the Satellite Era. Journal of Climate.

[13] Hegerl GC (2014). Challenges in Quantifying Changes in the Global Water Cycle. Bulletin of the American Meteorological Society.

[14] Yu L (2017). The Global Ocean Water Cycle in Atmospheric Reanalysis, Satellite, and Ocean Salinity. Journal of Climate.

[15] Wüst, G. Oberflächensalzgehalt, Verdunstung und Niederschlag auf dem Weltmeere. Länderkundliche Forschung, Festschrift Norbert Krebs. 347–359 (1936).

[16] Durack PJ, Wijffels SE, Matear RJ (2012). Ocean Salinities Reveal Strong Global Water Cycle Intensification During 1950 to 2000. Science.

[17] Skliris N (2014). Salinity changes in the World Ocean since 1950 in relation to changing surface freshwater fluxes. Climate Dynamics.

[18] Skliris N, Zika JD, Nurser G, Josey SA, Marsh R (2016). Global water cycle amplifying at less than the Clausius-Clapeyron rate. Scientific Reports.

[19] Terray L (2011). Near-Surface Salinity as Nature’s Rain Gauge to Detect Human Influence on the Tropical Water Cycle. Journal of Climate.

[20] Vinogradova NT, Ponte RM (2017). In Search of Fingerprints of the Recent Intensification of the Ocean Water Cycle. Journal of Climate.

[21] Buckley MW, Marshall J (2016). Observations, inferences, and mechanisms of the Atlantic Meridional Overturning Circulation: A review. Reviews of Geophysics.

[22] Boyer, T. P., Levitus, S., Antonov, J. I., Locarnini, R. A. & Garcia, H. E. Linear trends in salinity for the World Ocean, 1955–1998. *Geophysical Research Letters* **32**, 10.1029/2004GL021791 (2005).

[23] Durack PJ, Wijffels SE (2010). Fifty-Year Trends in Global Ocean Salinities and Their Relationship to Broad-Scale Warming. Journal of Climate.

[24] Hosoda S, Suga T, Shikama N, Mizuno K (2009). Global surface layer salinity change detected by Argo and its implication for hydrological cycle intensification. Journal of Oceanography.

[25] Singh HKA, Donohoe A, Bitz CM, Nusbaumer J, Noone DC (2016). Greater aerial moisture transport distances with warming amplify interbasin salinity contrasts. Geophysical Research Letters.

[26] Schmittner A, Silva TAM, Fraedrich K, Kirk E, Lunkeit F (2010). Effects of Mountains and Ice Sheets on Global Ocean Circulation. Journal of Climate.

[27] Marsh, R., Hazeleger, W., Yool, A. & Rohling, E. J. Stability of the thermohaline circulation under millennial CO2 forcing and two alternative controls on Atlantic salinity. *Geophysical Research Letters* **34**, 10.1029/2006GL027815 (2007).

[28] Seidov, D. & Haupt, B. J. On the role of inter-basin surface salinity contrasts in global ocean circulation. *Geophysical Research Letters* **29**, 10.1029/2002GL014813 (2002).

[29] Seidov, D. & Haupt, B. J. How to run a minimalist’s global ocean conveyor. *Geophysical Research Letters* **32**, 10.1029/2005GL022559 (2005).

[30] Stouffer RJ (2006). Investigating the Causes of the Response of the Thermohaline Circulation to Past and Future Climate Changes. Journal of Climate.

[31] Li, L., Schmitt, R. W., Ummenhofer, C. C. & Karnauskas, K. B. North Atlantic salinity as a predictor of Sahel rainfall. *Science Advances* **2** (2016). 10.1126/sciadv.1501588 PMC4928933 27386525

[32] Li L, Schmitt RW, Ummenhofer CC, Karnauskas KB (2016). Implications of North Atlantic Sea Surface Salinity for Summer Precipitation over the U.S. Midwest: Mechanisms and Predictive Value. Journal of Climate.

[33] Bengtsson L, Hodges KI, Roeckner E (2006). Storm Tracks and Climate Change. Journal of Climate.

[34] Chang EKM, Lee S, Swanson KL (2002). Storm Track Dynamics. Journal of Climate.

[35] Hoskins BJ, Hodges KI (2002). New Perspectives on the Northern Hemisphere Winter Storm Tracks. Journal of the Atmospheric Sciences.

[36] Shaw TA (2016). Storm track processes and the opposing influences of climate change. Nature Geosci.

[37] Ramos AM (2016). Atmospheric rivers moisture sources from a Lagrangian perspective. Earth Syst. Dynam..

[38] Eiras-Barca J, Brands S, Miguez-Macho G (2016). Seasonal variations in North Atlantic atmospheric river activity and associations with anomalous precipitation over the Iberian Atlantic Margin. Journal of Geophysical Research: Atmospheres.

[39] Yu, L., Jin, X. & Weller, R. A. Multidecade Global Flux Datasets from the Objectively Analyzed Air-sea Fluxes (OAFlux) Project: Latent and sensible heat fluxes, ocean evaporation, and related surface meteorological variables. Woods Hole Oceanographic Institution, Woods Hole, Massachusetts (2008).

[40] Adler RF (2003). The Version-2 Global Precipitation Climatology Project (GPCP) Monthly Precipitation Analysis (1979–Present). Journal of Hydrometeorology.

[41] Byrne MP, O’Gorman PA (2015). The Response of Precipitation Minus Evapotranspiration to Climate Warming: Why the “Wet-Get-Wetter, Dry-Get-Drier” Scaling Does Not Hold over Land. Journal of Climate.

[42] Yu, L. A global relationship between the ocean water cycle and near-surface salinity. *Journal of Geophysical Research: Oceans* **116**, 10.1029/2010JC006937 (2011).

[43] Gelaro R (2017). The Modern-Era Retrospective Analysis for Research and Applications, Version 2 (MERRA-2). Journal of Climate.

[44] Boyer, T. P. *et al*. World Ocean Database 2013. (ed S. Levitus) NOAA Atlas NESDIS 72 (2013).

[45] de Boyer Montégut, C., Madec, G., Fischer Albert, S., Lazar, A. & Iudicone, D. Mixed layer depth over the global ocean: An examination of profile data and a profile-based climatology. *Journal of Geophysical Research: Oceans* **109**, 10.1029/2004JC002378 (2004).

[46] Dee DP (2011). The ERA-Interim reanalysis: configuration and performance of the data assimilation system. Quarterly Journal of the Royal Meteorological Society.

[47] Kalnay E (1996). The NCEP/NCAR 40-Year Reanalysis Project. Bulletin of the American Meteorological Society.

[48] Large WG, Yeager SG (2009). The global climatology of an interannually varying air–sea flux data set. Climate Dynamics.

[49] Ren L, Hackert E, Arkin P, Busalacchi Antonio J (2014). Estimating the global oceanic net freshwater flux from Argo and comparing it with satellite-based freshwater flux products. Journal of Geophysical Research: Oceans.

[50] Bosilovich MG, Robertson FR, Takacs L, Molod A, Mocko D (2016). Atmospheric Water Balance and Variability in the MERRA-2 Reanalysis. Journal of Climate.

[51] Uppala SM (2006). The ERA-40 re-analysis. Quarterly Journal of the Royal Meteorological Society.

