# Supplementary material for: Water Vapor Transfer and Near-Surface Salinity Contrasts in the North Atlantic Ocean
Source: Sci Rep. 2018 Jun 11;8:8830. doi: 10.1038/s41598-018-27052-6 (PMC5995860; doi:10.1038/s41598-018-27052-6)
Supplement: Supplementary file 1 — Supplementary Information [file 41598_2018_27052_MOESM1_ESM.docx]

**Supplementary Information**

**Water Vapor Transfer and Near-Surface Salinity Contrasts in the North Atlantic Ocean**

James Reagan^1,2*^, Dan Seidov^2^, Tim Boyer^2^

^1^Earth System Science Interdisciplinary Center, University of Maryland, College Park, MD, USA

^2^National Centers for Environmental Information, NOAA, Silver Spring, MD, USA

*Corresponding Author: james.reagan@noaa.gov

**
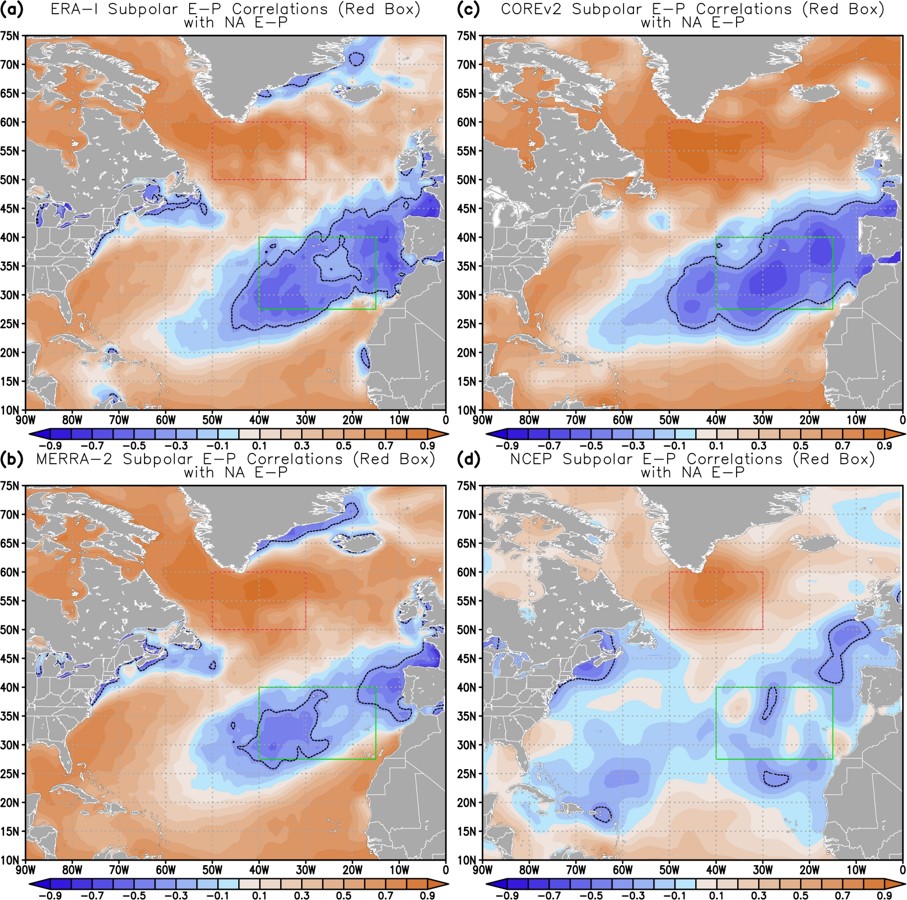
Supplementary Figure S1:** The 1985-2012 North Atlantic correlation between the area-averaged subpolar gyre E-P (red-contoured rectangle) and the E-P over the rest of the North Atlantic Ocean for a) ERA-I reanalysis, b) MERRA-2 reanalysis, c) COREv2, and d) NCEP/NCAR reanalysis. Shaded correlations are based on the 1985-1994, 1995-2004, and 2005-2012 monthly climatological E and P fields (N=36). The black dotted line represents the region where correlation is lower than -0.330 (95% CI). The red-contoured box has the following boundaries: 310-330°E and 50-60°N. This figure is similar to Fig. 2b in the main text. This figure was created using the Grid Analysis and Display System (GrADS) software (available at: http://cola.gmu.edu/grads/).

**Supplementary Figure S2:** The 1985-2012 North Atlantic time series of area-averaged E-P over the subpolar NA (red box in Supp. Fig. S1) and E-P over the subtropical NA (green box in Supp. Fig. S1) for a) ERA-I reanalysis, b) MERRA-2 reanalysis, c) COREv2, and d) NCEP/NCAR reanalysis. Time series and corresponding correlation between the two time series in each plot is based on the 1985-1994, 1995-2004, and 2005-2012 monthly climatological E and P fields (N=36). The red box in Supp. Fig. S1 has the boundaries: 310-330°E and 50-60°N and the green box has the boundaries: 320-345°E and 27.5-40°N. This figure is similar to Fig. 2c in the main text. This figure was created using the GrADS software.

**
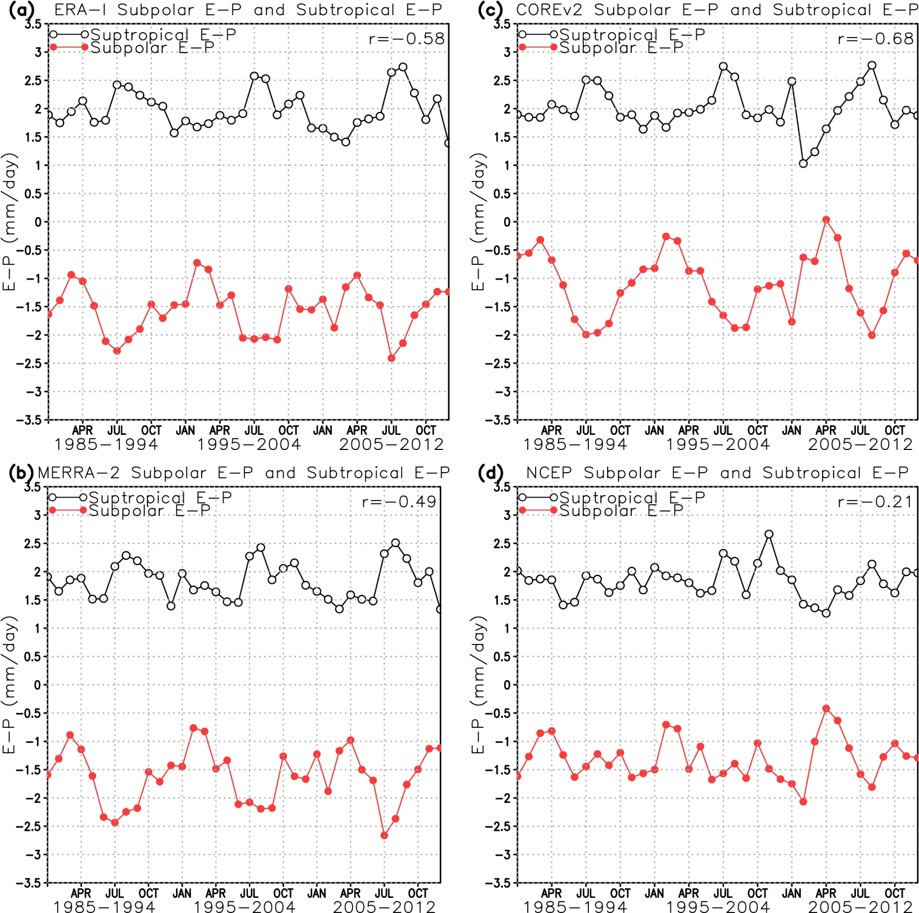
**

**Supplementary Figure S3:** Similar to Supp. Fig S1, but represents correlations between area-average subpolar NSS (red box) and NA E-P for a) ERA-I reanalysis, b) MERRA-2 reanalysis, c) COREv2, and d) NCEP/NCAR reanalysis. The black dotted line represents the region where correlation is lower than -0.330 (95% CI). Boundaries remain the same from Supp. Fig. S1. This figure is similar to Fig. 3a in the main text. This figure was created using the GrADS software.

**
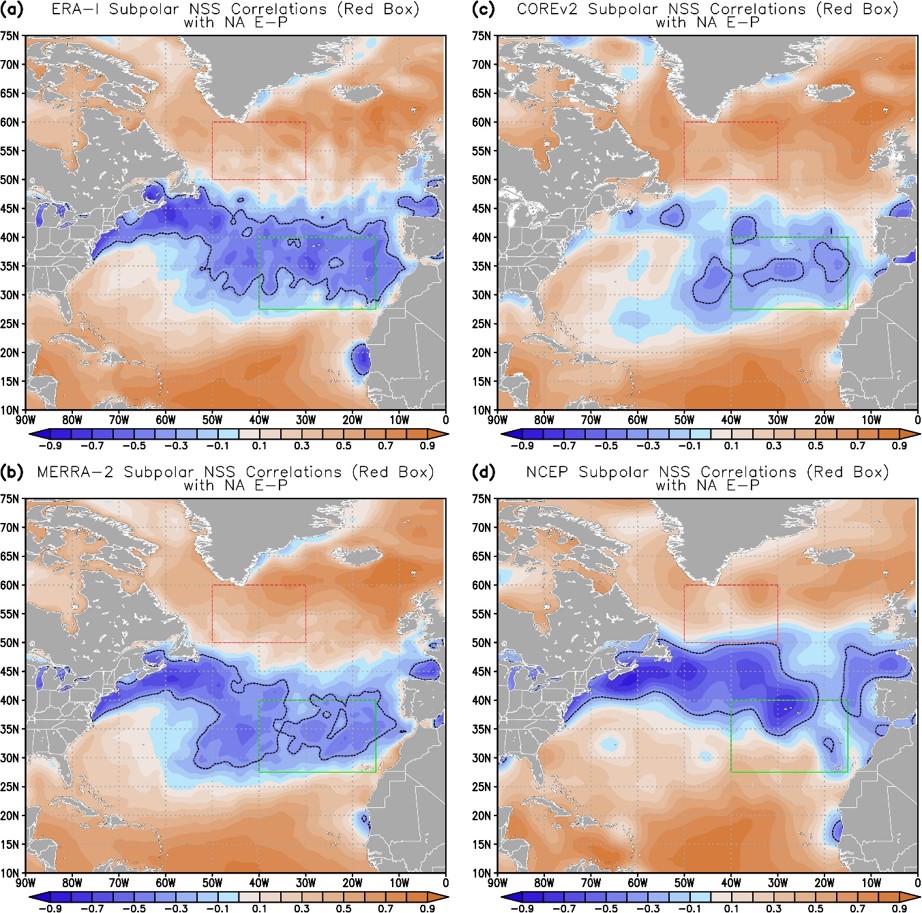
**

**Supplementary Figure S4:** Similar to Supp. Fig S2, but represents the time series of area-averaged NSS over the subpolar NA (red box in Supp. Fig. S3) and E-P over the subtropical NA (green box in Supp. Fig. S3) for a) ERA-I reanalysis, b) MERRA-2 reanalysis, c) COREv2, and d) NCEP/NCAR reanalysis. Boundaries remain the same from Fig. S2. This figure is similar to Fig. 3b in the main text. This figure was created using the GrADS software.


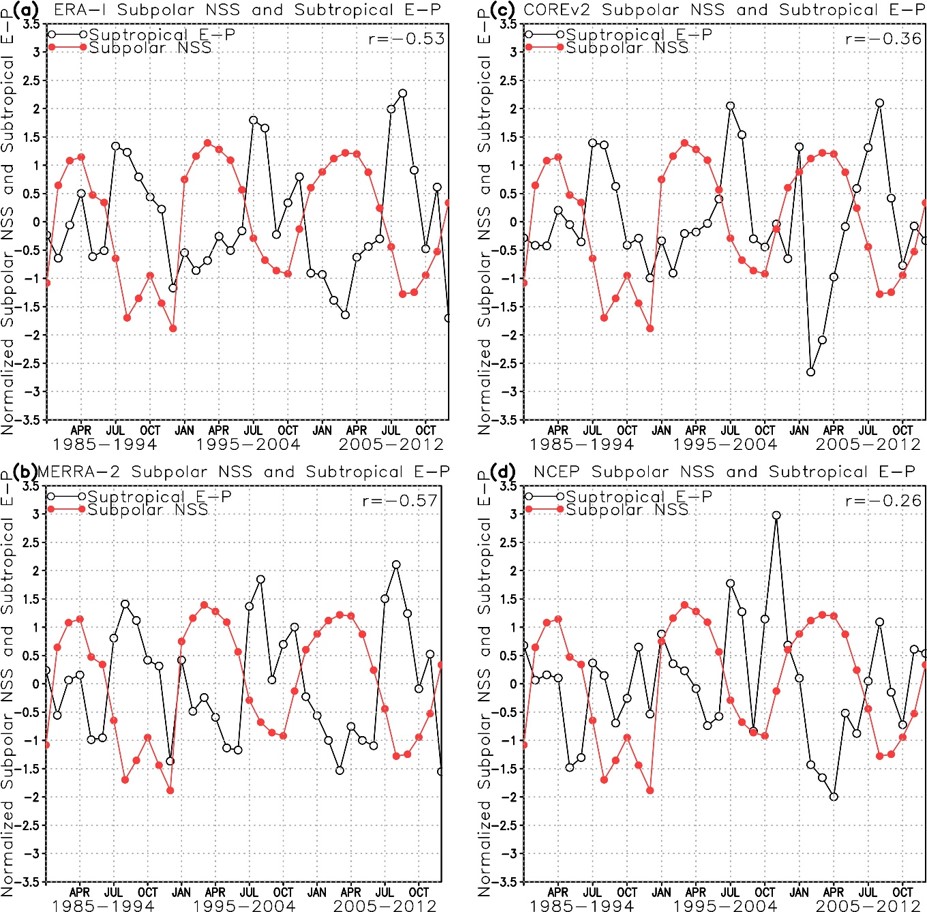


**Supplementary Figure S5:** The 1985-2012 seasonal average of the vertically integrated moisture flux divergence (VIMFD, mm*day^-1^, shaded) and the divergent component of the moisture fluxes (DCMF, kg*m^-1^*sec^-1^, vectors) for ERA-I reanalysis during a) winter (JFM), b) spring (AMJ), c) summer (JAS), and d) fall (OND). Orange shades represent moisture divergence and blue shades represent moisture convergence. This is similar to Fig. 4 in the main text. This figure was created using the GrADS software.


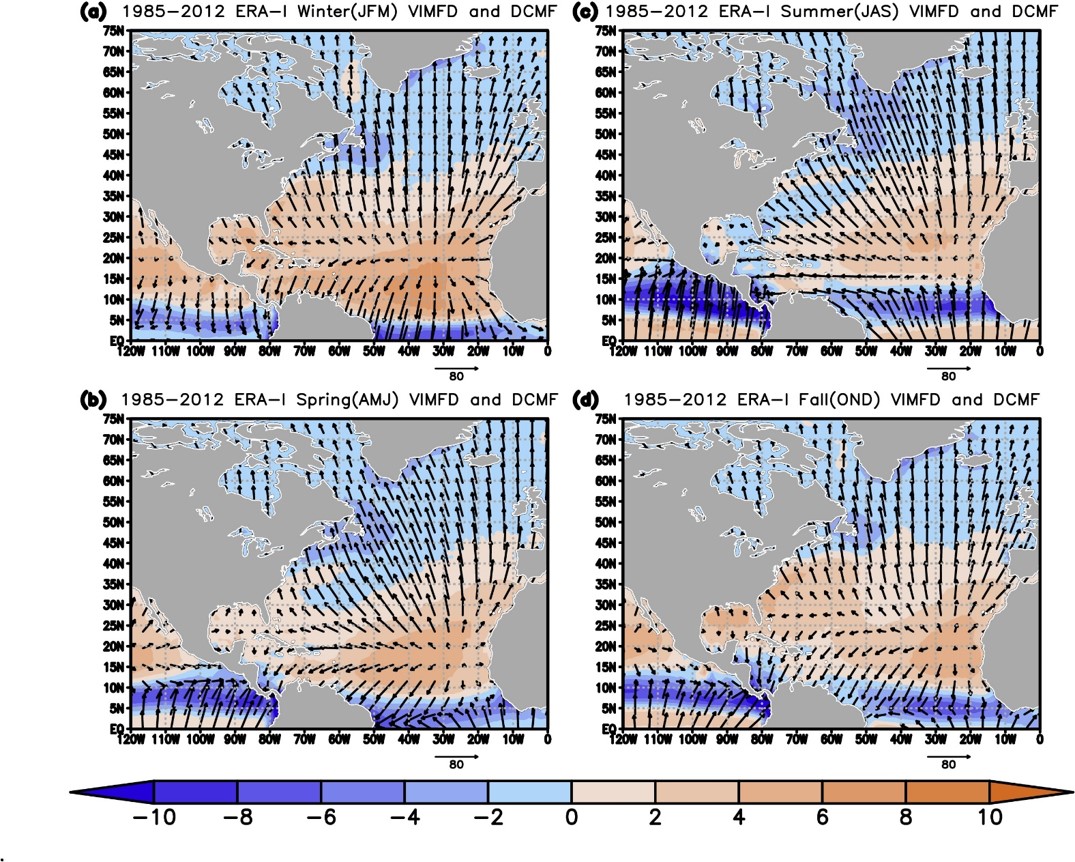


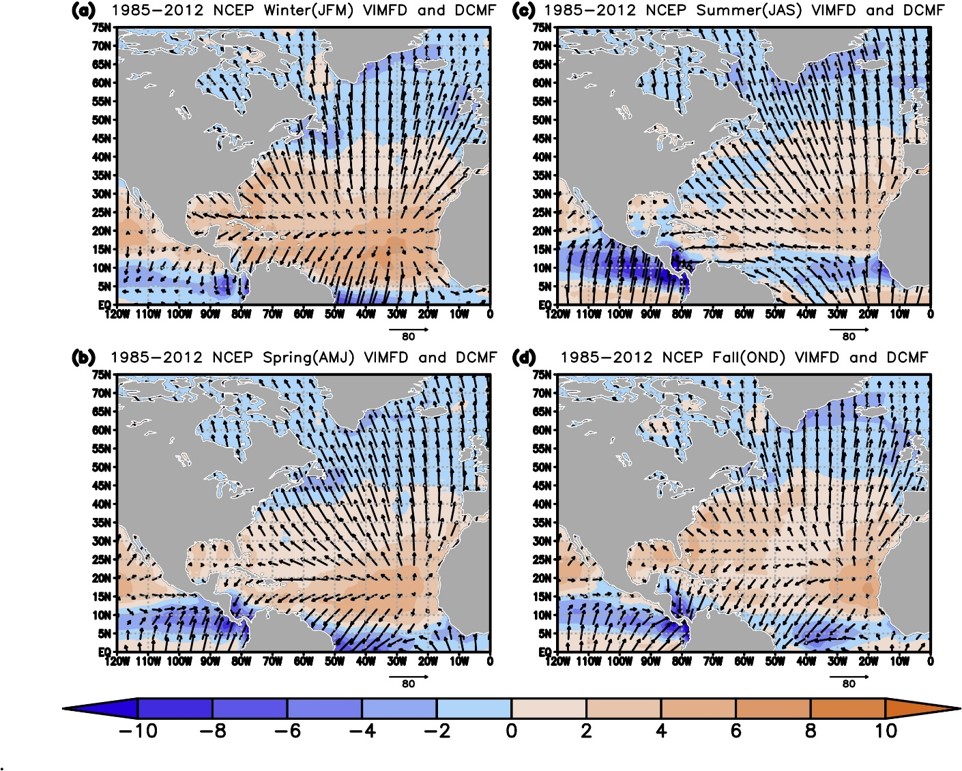
**Supplementary Figure S6:** The 1985-2012 seasonal average of the vertically integrated moisture flux divergence (VIMFD, mm*day^-1^, shaded) and the divergent component of the moisture fluxes (DCMF, kg*m^-1^*sec^-1^, vectors) for NCEP/NCAR reanalysis during a) winter (JFM), b) spring (AMJ), c) summer (JAS), and d) fall (OND). Orange shades represent moisture divergence and blue shades represent moisture convergence. This is similar to Fig. 4 in the main text. This figure was created using the GrADS software.

**Supplementary Figure S7:** The 1985-2012 zonal average of the meridional component of the DCMF (kg*m^-1^*sec^-1^, black line) and of the VIMFD (10*mm*day^-1^, red line) for ERA-I reanalysis during a) winter (JFM), b) spring (AMJ), c) summer (JAS), and d) fall (OND). The zonal average was taken over the 60°W-20°W area (see Supp. Fig. S5a-d). This is similar to Fig. 5 in the main text. This figure was created using the GrADS software.


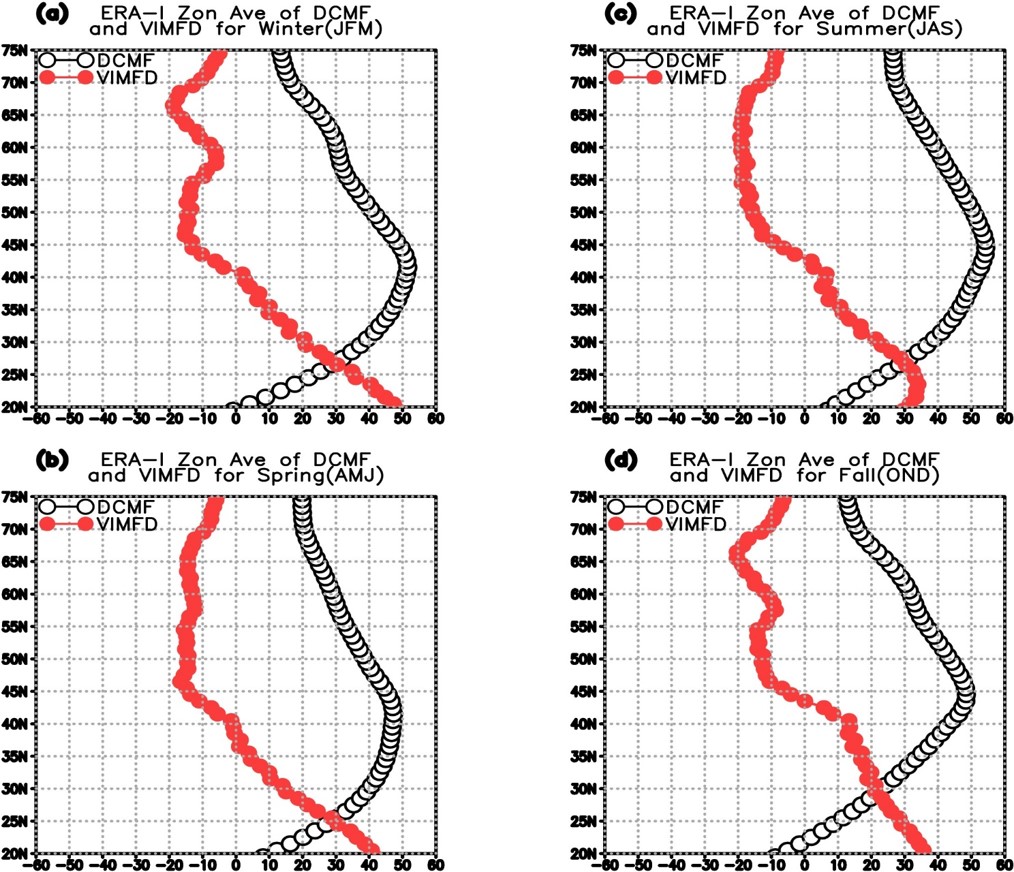


**Supplementary Figure S8:** The 1985-2012 zonal average of the meridional component of the DCMF (kg*m^-1^*sec^-1^, black line) and of the VIMFD (10*mm*day^-1^, red line) for NCEP/NCAR reanalysis during a) winter (JFM), b) spring (AMJ), c) summer (JAS), and d) fall (OND). The zonal average was taken over the 60°W-20°W area (see Supp. Fig. S6a-d). This is similar to Fig. 5 in the main text. This figure was created using the GrADS software.


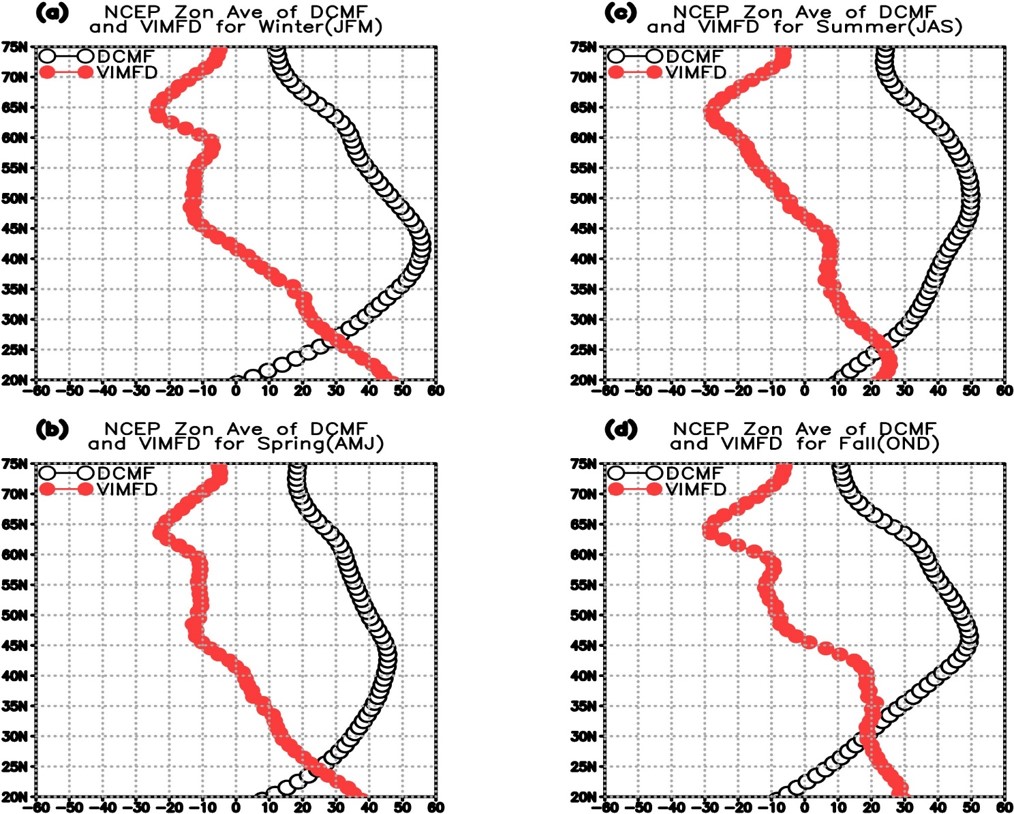


**Supplementary Figure S9:** Area-average E-P for five monthly decadal averages from 1957-2002 calculated from the ERA-40 reanalysis over the subpolar NA (red line, boundaries: 310-330°E and 50-60°N) and subtropical NA (black line, boundaries: 320-345°E and 27.5-40°N). Note the degraded seasonal E-P signal in the subpolar NA for the 1957-64 and 1965-1974 decades which is likely due to poor representation of precipitation in the ERA-40 reanalysis in the subpolar NA during this time. The seasonal signal improves once satellite data begins to be assimilated (~1979) into the ERA-40 reanalysis. This figure was created using the GrADS software.

**
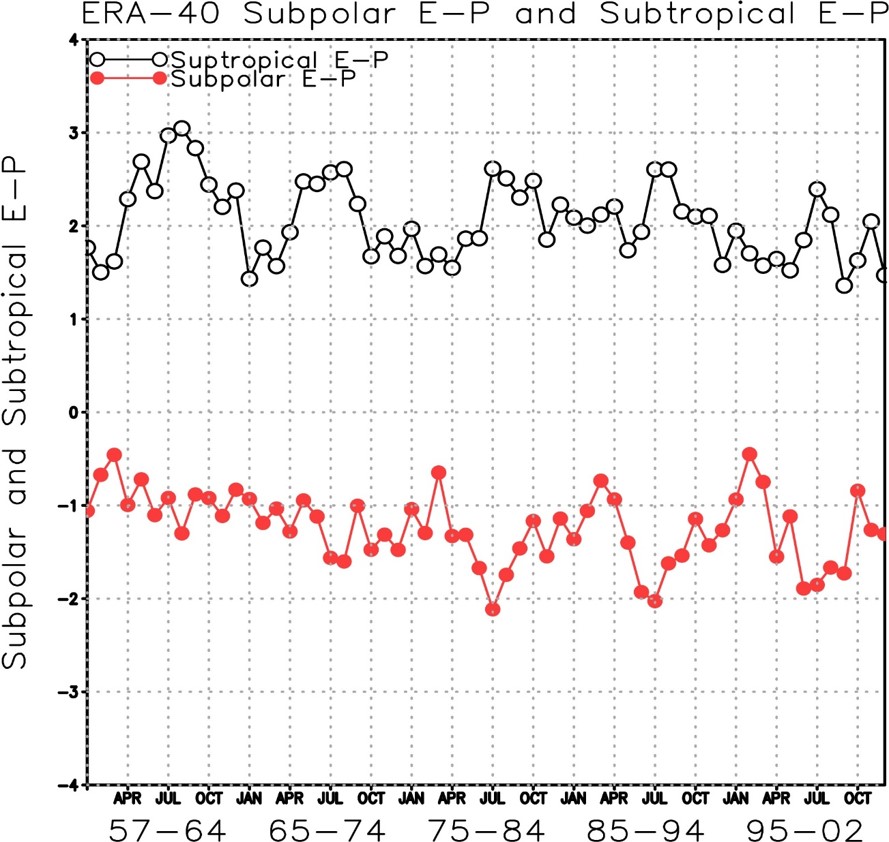
**
